# The insular cortex is not insular in thyroid eye disease: neuroimaging revelations of central–peripheral system interaction

**DOI:** 10.1186/s12974-024-03044-4

**Published:** 2024-02-17

**Authors:** Haiyang Zhang, Yuting Liu, Duojin Xia, Mengda Jiang, Yinwei Li, Jing Sun, Haixia Guan, Ling Zhu, Xuefei Song, Jue Wang, Xianqun Fan, Huifang Zhou

**Affiliations:** 1 Department of Ophthalmology, Shanghai Ninth People’s Hospital, Shanghai Jiao Tong University School of Medicine, Shanghai, China; 2 Shanghai Key Laboratory of Orbital Diseases and Ocular Oncology, Shanghai, China; 3 School of Health Science and Engineering, University of Shanghai for Science and Technology, Shanghai, China; 4 Department of Radiology, Shanghai Ninth People’s Hospital, Shanghai Jiao Tong University School of Medicine, Shanghai, China; 5 Department of Endocrinology, Guangdong Provincial People’s Hospital (Guangdong Academy of Medical Sciences), Southern Medical University, Guangzhou, China; 6 Institute of Sports Medicine and Health, Chengdu Sport University, Chengdu, China

**Keywords:** Functional connectivity, Functional magnetic resonance imaging, Insular cortex, Neuroimmunomodulation, Thyroid eye disease

## Abstract

**Background:**

Thyroid eye disease (TED) is highly correlated with dysregulated immunoendocrine status. The insular cortex was found to regulate peripheral inflammation and immunomodulation in mice. This study aimed to explore whether the insular cortex in patients with TED played a modulatory role including the aberrant brain functional alteration and its association with immunoendocrine status.

**Methods:**

This study included 34 active patients (AP), 30 inactive patients (IP) with TED, and 45 healthy controls (HC) matched for age, sex, and educational level. Comprehensive clinical details (especially immunoendocrine markers) and resting-state functional magnetic resonance imaging data were collected from each participant. The amplitude of low-frequency fluctuation (ALFF) was used to probe the aberrant alterations of local neural activity. The seed-based functional connectivity (FC) analysis was used to explore the relationship between the insular cortex and each voxel throughout the whole brain. The correlation analysis was conducted to assess the association between insular neurobiomarkers and immunoendocrine parameters.

**Results:**

When compared with the IP and HC groups, the AP group displayed significantly higher ALFF values in the right insular cortex (INS.R) and lower FC values between the INS.R and the bilateral cerebellum. None of the neurobiomarkers differed between the IP and HC groups. Besides, correlations between insular neurobiomarkers and immunoendocrine markers (free thyroxine, the proportion of T cells, and natural killer cells) were identified in both AP and IP groups.

**Conclusions:**

This study was novel in reporting that the dysregulation of the insular cortex activity in TED was associated with abnormal peripheral immunoendocrine status. The insular cortex might play a key role in central–peripheral system interaction in TED. Further research is crucial to enhance our understanding of the central–peripheral system interaction mechanisms involved in autoimmune diseases.

**Supplementary Information:**

The online version contains supplementary material available at 10.1186/s12974-024-03044-4.

## Introduction

The aberrant immunoendocrine status observed in thyroid eye disease (TED), an autoimmune condition frequently secondary to Graves’ disease, is characterized by the presence of abnormal autoimmune antibodies and infiltration of lymphocytes in the peripheral blood [1, 2]. This immunopathological state leads to endocrinopathy, including thyroid hormone disturbance and the involvement of orbital soft tissues, contributing to the development of distinct clinical manifestations including proptosis, diplopia, vision loss, and so forth [3–5]. These debilitating symptoms significantly impact the quality of life of patients [6]. TED can be categorized into two stages based on the intensity of immune responses: the active stage characterized by heightened immune activity and the inactive stage marked by relatively subdued reactions [7]. Although these stages exhibit different patterns of immunoendocrine statuses in TED, the underlying immunomodulation mechanisms remain unclear.

Modulation of abnormal immunoendocrine status poses significant challenges in managing autoimmune diseases such as TED [8]. Traditional immunomodulation strategies, including peripheral administration of immune modulators and anti-inflammatory drugs, have limited efficacy in complex autoimmune diseases [9, 10]. Recent studies investigated the modulating role of the central system in the peripheral immunity system [11]. It was suggested that peripheral immunopathology could trigger alterations in the central system with immunity regulation [12], and various peripheral autoimmune diseases were found to exhibit changes in the central system.

Recent studies shed light on the involvement of the central system in the comprehensive modulation of immunity, with special attention to the insular cortex [13, 14]. Specifically, a recent study uncovered a significant association between the insular cortex and the peripheral immune system [15]. The study demonstrated that the insular cortex contained cells with memory traces of responses to peripheral immune reactions and could reactivate these reactions, emphasizing its crucial role in neuroimmunomodulation. In the emerging field of neuroimaging on TED, the involvement of the insular cortex has been observed, prompting us to direct our attention toward this area [16, 17].

TED is an autoimmune disease, and hence it is crucial to advance our understanding of the relationship between the insular cortex and peripheral immunoendocrine status in TED at a clinical level. We hypothesized that dysregulated insular cortex activity might exist in patients with TED and vary among different immunoendocrine statuses. Therefore, our study involves a comprehensive analysis employing neuroimaging and immunoendocrine profiling for patients with varying immunoendocrine statuses of TED, aiming for more definitive verification. Resting-state functional magnetic resonance imaging (rs-fMRI) was employed to measure local neural activity and the association between brain regions. Specifically, we focused on the amplitude of low-frequency fluctuation (ALFF) to detect any aberrant changes in local neural activity and on the functional connectivity (FC) matrix to identify shifts in the brain network and spatiotemporal interconnections between brain regions in the resting state [18, 19]. Currently, only a few studies have utilized ALFF or FC analysis in TED [20–24]. All of them investigated alterations at the whole-brain level rather than focusing on specific regions. We hypothesized that, at the whole-brain level, the insula may exhibit altered activity in this autoimmune disease. Using this area as a seed for further investigation, we anticipate changes in its connectivity. Furthermore, we incorporated immunoendocrine markers, such as thyroid hormones and the cluster of differentiation (CD), aiming to elucidate patients' peripheral immunoendocrine statuses. These markers serve as indicators, reflecting both disease severity and the patients' cell-mediated immune responses [25]. By conducting correlations between neurobiomarkers and immunoendocrine markers, we aimed to shed more light on the crucial role of the insular cortex in the central–peripheral system interactions of TED.

## Materials and methods

### Study participants

**Fig. 1:**
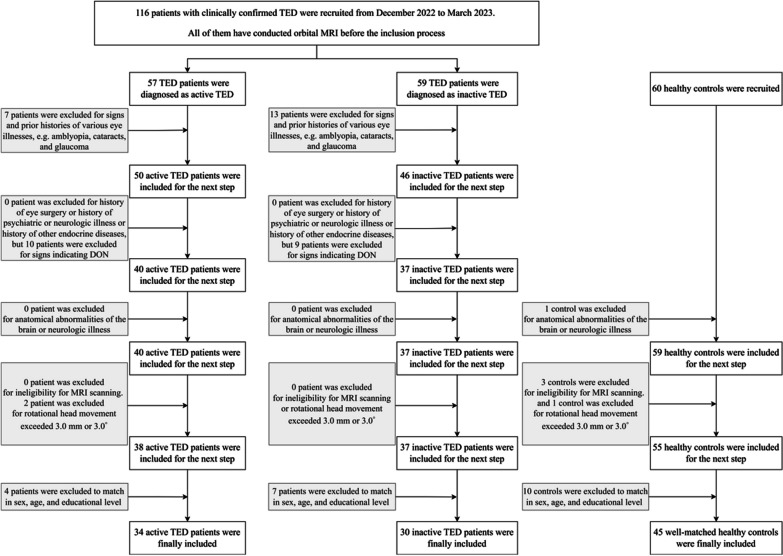
Flowchart of the inclusion process of our study and follow-up analysis. TED: thyroid eye disease; MRI: magnetic resonance imaging

According to the clinical guidelines proposed by the European Group on Graves' Orbitopathy (EUGOGO) [26], 116 patients with TED, including 57 active patients (AP) and 59 inactive patients (IP), were recruited, along with 60 HC well matched in terms of sex, age, and education level. Disease duration was determined from the onset of ocular manifestations. TED activity was evaluated based on a combination of the seven-point clinical activity score (CAS) and orbital MRI, adhering to clinical guidelines [26, 27]. Detailed information on patients and exclusion criteria are displayed in Additional file 1: Material S1. (Fig. 1).

### Immunoendocrine status assessment

As discussed earlier, patients with TED were divided into two groups with different immunoendocrine statuses (AP and IP) based on their disease activity. This was assessed by combining the seven-point CAS and orbital MRI findings. The AP and IP groups exhibited significant differences in immunoendocrine status and displayed distinct immunomodulation patterns. The clinical laboratory conducted a comprehensive set of tests, including serum thyroid function tests [thyroid-stimulating hormone (TSH), free triiodothyronine (fT3), and free thyroxine (fT4)], thyroid antibody tests [TSH receptor antibody (TRAb)], and lymphocyte subset analyses [CD3^+^ T cells, CD19^+^ B cells, and CD16^+^CD56^+^ natural killer (NK) cells]. Further details are presented in Additional file 1: Material S2.

### Neuroimaging assessment

Neuroimaging acquisition involved performing wakeful rs-fMRI was conducted on a 3 T scan using a 64-channel phased-array head coil (Magnetom Vida, Siemens, Erlangen, Germany). All subjects were required to close their eyes without dozing off when undergoing MRI scanning to reduce the interference of vigilance [28]. The DPABI version 7.0 (http://rfmri.org/DPABI) was used to preprocess all of the rs-fMRI data based on the MATLAB platform (www.mathworks.com/products/matlab) for neuroimaging data processing [29]. For the purposes of magnetization balancing, the first 10 functional volumes were eliminated. Slice timing and realignment for head motion correction were performed. Images were normalized to the Montreal Neurological Institute EPI template (resampling voxel size = 3 × 3 × 3 mm^3^). A 6-mm full-width at half-maximum Gaussian kernel was applied for smoothing. Detrending was applied to remove linear trends. Finally, the nuisance covariates were eliminated using linear regression, including the average signals from the cerebrospinal fluid and white matter as well as the six head motion parameters. For the calculation of FC, head motion scrubbing regressors [framewise displacement (FD) threshold 0.2 for “bad” time] were also added in the linear regression to address the concern of motion [30–32].

Three participants were excluded from the analysis due to their maximal translational or rotational head movements exceeding 3.0 mm or 3.0^◦^. Moreover, there were no differences among the three groups (p = 0.1754) in FD, and the mean FD of the AP, IP, and HC group was 0.17, 0.17, and 0.14, respectively [33].

The ALFF and FC were computed across the whole brain. Time courses were converted into the frequency domain using a fast Fourier transform algorithm, enabling each voxel to represent the amplitude of the signal across the whole spectrum. The averaged square root of the spectrum spanning the frequency range of 0.01–0.08 Hz was considered as the ALFF measurement. The mean ALFF was obtained as the ALFF value divided by the global mean ALFF value for standardized variability among the participants.

After the ALFF analysis, the cluster of the insular cortex identified from the ALFF analysis was saved as the seed to delve deeper into the brain functional network via whole-brain FC analysis in a voxel-wise whole-brain manner. Fisher’s *Z* transformation was then performed for normalization and statistical analysis. Further details are presented in Additional file 1: Material S3.

### Statistical analyses

A total of 64 patients with TED, including 34 AP and 30 IP, along with 45 well-matched HC, were included for statistical analyses finally. The demographic and clinical data were analyzed using GraphPad Prism 9 (GraphPad, CA, USA). To compare the differences among the AP, IP, and HC groups, the one-way analysis of variance (ANOVA) test was conducted for continuous variables with normal distribution and Kruskal–Wallis tests were conducted for non-normally distributed data. The independent-sample *t* tests were conducted for continuous variables with a Gaussian distribution, whereas Mann–Whitney *U* tests were conducted for non-Gaussian distribution data. The chi-square tests were conducted for categorical variables. A *P* value < 0.05 indicated a statistically significant difference.

The DPABI toolbox 7.0 was used for analyzing ALFF and FC between the three groups [29]. ANOVA was conducted to compare the group differences in the ALFF and FC values. The post hoc two-sample *t* tests were conducted to compare the values in brain regions with significant differences between each pair of groups (AP vs. IP, AP vs. HC, IP vs. IP). Multiple comparison correction was conducted within the whole brain, and significant clusters were identified using a voxel-level threshold of *P* < 0.001 and a cluster-level threshold of *P* < 0.05 [Gaussian random field (GRF) correction] with a two-tailed test. BrainNet Viewer was used to visualize connectivity in a brain model [34].

The relationship between the immunoendocrine markers and neurobiomarkers in regions with significant group differences was assessed using linear regression models. The threshold for statistical two-tailed significance was set at *P* < 0.05. Finally, receiver operating characteristic curve analysis was conducted on the same discovery sample.

## Results

### Alterations in insular neurobiomarkers in TED

**Fig. 2:**
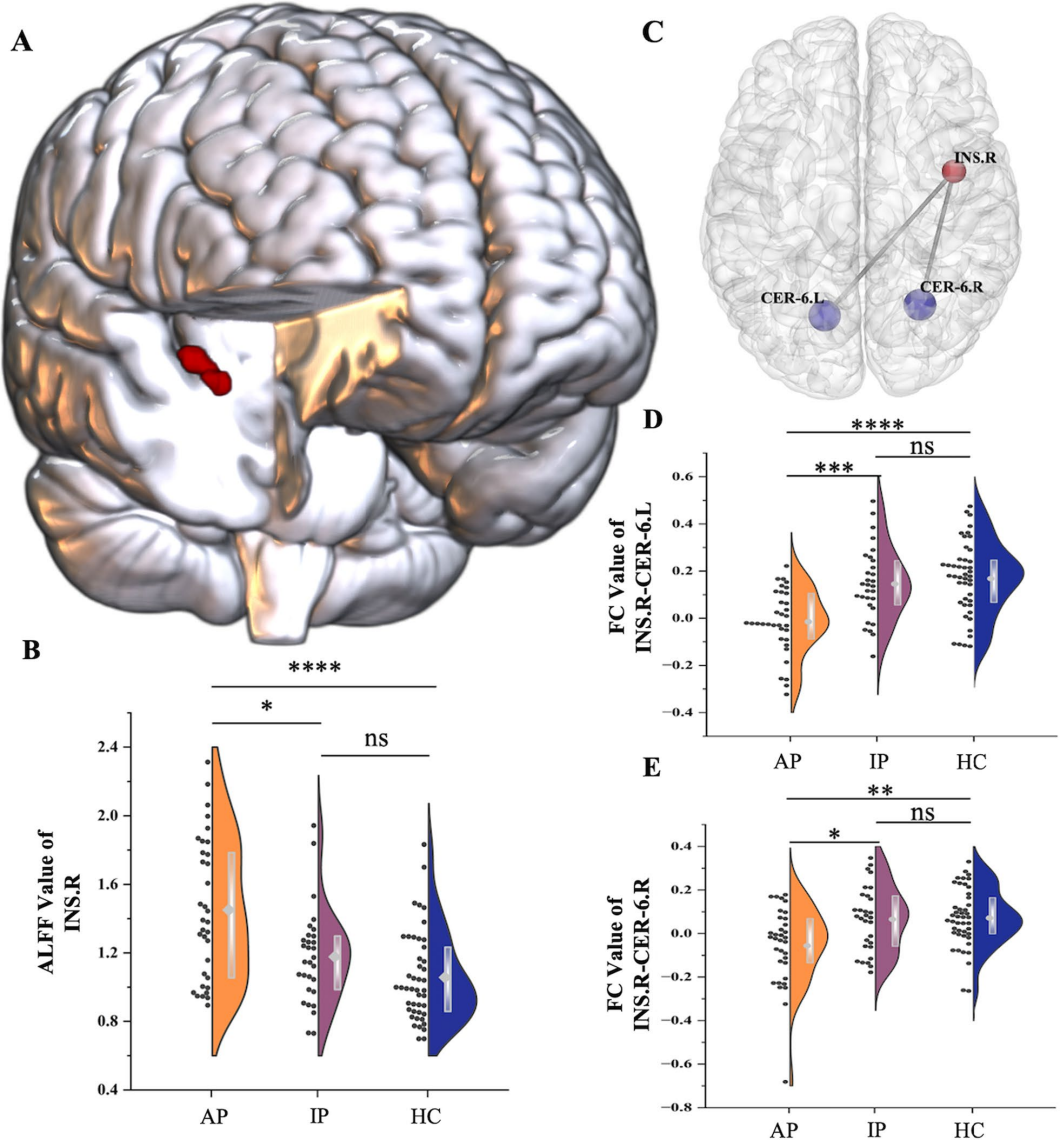
Changes in insular neurobiomarkers and differences between groups. **A** Elevated insular cortex activity was observed during the resting state in patients with thyroid eye disease with different immunoendocrine statuses compared with controls (voxel-level *P* < 0.001, cluster-level *P* < 0.05, GRF correction). **B** Violin and box plots demonstrated the ALFF differences of the INS.R in the AP, IP, and HC groups. **C** Decreased FC between the INS.R and the right cerebellum was found in patients with thyroid eye disease with different immunoendocrine statuses compared with controls. **D**, **E** Group differences among the AP, IP, and HC groups. The differences primarily existed in the CER.L and CER.R (voxel-level *P* < 0.001, cluster-level *P* < 0.05, GRF correction). The violin and box plot demonstrated the FC differences of CER.L and CER.R in the AP, IP, and HC groups. Ns, *P* > 0.05; ^*^P < 0.05; ^**^P < 0.01; ^***^P < 0.001; ^****^P < 0.0001. ALFF: amplitude of low-frequency fluctuations; AP: active patients; CER-6.L: left cerebellum 6; CER-6.R: right cerebellum 6; FC: functional connectivity; HC: healthy controls; INS.R: right insular cortex; IP: inactive patients

Tables 1, 2, and Additional file 1: Material S4 present the demographic and clinical characteristics and the differences in immunoendocrine markers. The results of the one-way ANOVA found that the brain activity of the right insular cortex (INS.R) and other brain regions (left inferior parietal lobule and right paracentral lobule) were significantly different among the three groups. The activity of the insula was increased only in AP, while no significant difference was found between IP and HC. The INS.R was selected as the seed. The result of voxel-wise whole-brain FC analysis showed significantly lower FC values with the left and right cerebellum 6 (CER-6.L and CER-6.R, respectively). Both FC values were significantly lower in AP compared with those in the IP group, while no difference was observed between IP and HC, (Fig. 2, Additional file 1: Table S1 and Material S5).

**Table 1:** Demographic and clinical characteristics of TED patients and controls

Characteristics	AP (n = 34)	IP (n = 30)	HC (n = 45)	*P* value	
				AP vs IP	AP vs IP vs HC
Sex, Male (n, %)	11 (32.3%)	9 (30%)	21 (46.7%)	0.839	0.257
Age (year)	46.85 ± 10.31	41.7 ± 12.75	42.93 ± 14.93	0.079	0.246
Years of education (year)	13.50 (11.25, 16.00)	16.00 (9.000, 16.75)	16.00 (12.00, 17.50)	0.339	0.488
Disease duration (month)	13 (7, 32)	15 (9, 35)	–	0.606	–
CAS	3.26 ± 1.32	0.93 ± 0.98	–	< 0.0001	–

The last column refers to the results of ANOVA

TED: thyroid eye disease; AP: active patients; IP: inactive patients; HC: healthy controls; CAS: clinical activity score

**Table 2:** Immunoendocrine markers differences of active and inactive TED patients

Characteristics	AP	IP	*P* value
TSH (mIU/L)	1.245 (0.298, 3.104)	1.480 (0.295, 3.210)	0.824
fT3 (pmol/L)	3.420 (3.090, 3.860)	3.890 (3.500, 4.790)	0.046
fT4 (pmol/L)	0.905 (0.763, 1.565)	10.57 (0.825, 17.67)	0.012
TRAb (IU/L)	9.220 (3.515, 29.05)	3.020 (1.310, 13.28)	0.004
CD3^+^ T cells	71.76 (67.72, 76.85)	74.73 (65.15, 76.95)	0.747
CD19^+^ B cells	14.90 (11.47, 16.96)	13.23 (10.02, 16.27)	0.271
CD16^+^CD56^+^ NK cells	11.21 (7.078, 16.98)	12.92 (9.045, 21.38)	0.633

TED: thyroid eye disease; AP: active patients; IP: inactive patients; HC: healthy controls; TSH: thyroid-stimulating hormone; fT3: free triiodothyronine; fT4: free thyroxine; TRAb: Thyroid-Stimulating Hormone Receptor Antibodies; CD: the cluster of differentiation

### Correlation between insular neurobiomarkers and immunoendocrine markers and the clinical relevance

**Fig. 3:**
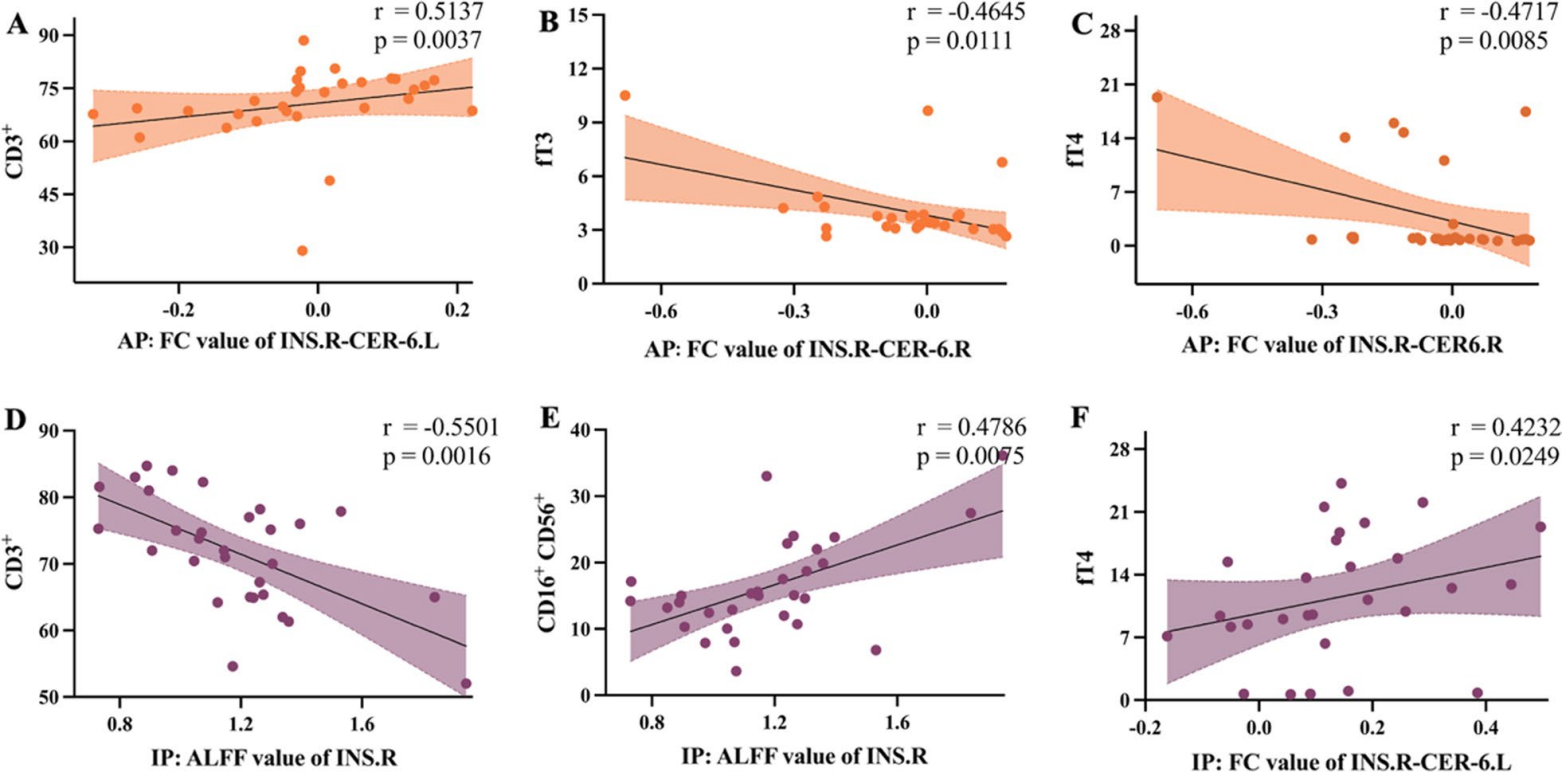
Correlation analysis results show relationships between neurobiomarkers and immunoendocrine markers. Black lines depict linear regression with a 95% confidence interval (shadow). *X*-axis: neurobiomarkers; *Y*-axis: immunoendocrine markers. ALFF: Amplitude of low-frequency fluctuations; CD: the cluster of differentiation; CER-6.L: left cerebellum-6; FC: functional connectivity; fT3: free triiodothyronine; fT4: free thyroxine; INS.R: right insular cortex

The correlation analyses were conducted in both AP and IP groups. Within the AP group, the insula–cerebellum FC correlated with CD3^+^ T cells, fT3, and fT4 (CD3^+^ T cells: *r* = 0.5317, *P* = 0.0037; fT3: *r* = − 0.4645, *P* = 0.0111; fT4: *r* = − 0.4717, *P* = 0.0085). Within the IP group, correlations were found between the ALFF of INS.R and CD3^+^ T cells (*r* = − 0.5501, *P* = 0.0016). The insula–cerebellum FC correlated with fT4 and CD16^+^CD56^+^ NK cells (fT4: *r* = 0.4232, *P* = 0.0249; CD16^+^CD56^+^ NK cells: *r* = 0.4786, *P* = 0.0075) (Fig. 3). No correlation was found between other insular neurobiomarkers and immunoendocrine markers. Despite not surviving the Bonferroni correction, the relationship in the AP group between the FC of INS.R-CER-6.L and CD3^+^ T cells, and the correlation in the IP group between ALFF of INS.R and CD3^+^ T cells remained significant even after correction across the 7 immunoendocrine markers.

**Fig. 4:**
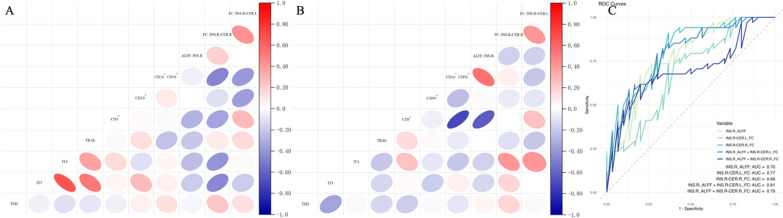
The correlation analysis and receiver operating characteristic curves for diagnosing the immunoendocrine status. **A** The correlation analysis results between all neurobiomarkers and immunoendocrine markers in AP. **B** The correlation analysis results between all neurobiomarkers and immunoendocrine markers in IP. **C** The receiver operating characteristic curves for diagnosing the immunoendocrine status of TED based on neurobiomarkers. The analysis revealed that the combination of activity of the insular cortex and the functional connectivity of insula-cerebellum demonstrated optimal diagnostic performance for immunoendocrine status (AUC = 0.81). ALFF: amplitude of low-frequency fluctuations; FC: functional connectivity; INS.R: right insular cortex; CER.L: left cerebellum 6; CER.R: right cerebellum 6; TSH: thyroid-stimulating hormone; fT3: free triiodothyronine; fT4: free thyroxine; TRAb: thyroid-stimulating hormone receptor antibody; CD: the cluster of differentiation; AUC: area under curve

Figure 4A and B depict the relationship between all neurobiomarkers and immunoendocrine markers in more detail with a heat map. Moreover, the performance of the neurobiomarkers related to the insular cortex for identifying different immunoendocrine statuses of TED was satisfying, with the combination of the activity of the insular cortex; the insula–cerebellum FC demonstrated optimal diagnostic performance for immunoendocrine status (AUC = 0.81) (Fig. 4C). The combination of the ALFF of INS.R and FC between INS.R and CER.L was the strongest predictor of the immunoendocrine status of TED.

## Discussion

This study validated our hypothesis regarding the correlation between insular cortex activity and immunoendocrine status in TED. A previous neuroimaging study reported elevated insular cortex activity in TED [17], but other studies did not draw the same conclusion probably due to the heterogeneity of TED and small sample size [22, 35]. In this setting, we found the animal-level study conducted by Koren et al. in 2021 [15], which revealed that peripheral immune reactions impacted the insula and created a memory trace of the immunomodulation in mice. This demonstrated the pivotal role of the insular cortex in neuroimmunomodulation. Therefore, we grouped patients with TED into active (AP) or inactive (IP) according to immunoendocrine status. Specifically, we observed significant dysregulation in the local activity of the insular cortex and insula–cerebellum connectivity in patients with active TED, whereas no significant difference was found between patients with inactive TED and HC. The correlation analysis between insular neurobiomarkers and immunoendocrine markers in TED further supported our hypothesis. This study was novel in investigating the central–peripheral system interaction in TED, which is a complex autoimmune disease, hence providing new avenues for understanding the underlying mechanisms at play.

The neuroimaging finding revealed that the insular cortex activity was reactive to active immunoendocrine status in TED. Besides, the insula–cerebellum connectivity was diminished in patients with active TED. The cerebellum regulates visuospatial perception [36]. Although its involvement in TED has not been fully investigated, the common visual dysfunctions that patients with TED encountered (especially diplopia) might include such experiences [37]. Besides, two recent studies have reported neuroimaging alterations in the cerebellum in TED [38, 39]. Our findings offered a potential new explanation for immune-related changes in the cerebellum and their associated visual dysfunctions. Moreover, the study conducted by Jia et al. [40] identified significant changes in connectivity between the insula and the cerebellum in inflammation-induced mouse models. These findings aligned with our proposed explanation at the animal level. However, it is essential to consider that the calculation of functional connectivity relies on the consistency of signals across different brain regions [18]. Therefore, alterations in the cerebellum might also be influenced by visual dysfunction in TED or other influencing factors. Consequently, further analyses and studies are required to investigate the causal relationship between the insular cortex and the cerebellum.

The group analysis did not reveal any significant difference in insular neurobiomarkers in patients with inactive TED compared with the HC. Nevertheless, this lack of significance might be attributed to the correction applied to small sample sizes, which limited the sensitivity of the analysis [41]. Both the local activity of the insular cortex and the insula–cerebellum connectivity were correlated with immunoendocrine markers during correlation analysis of inactive TED. This suggested that certain changes might have occurred in the insular cortex in patients with TED with subdued immune responses. With an improved understanding of the immune mechanism and subtyping of TED, we can gain deeper insights into the reasons behind these observed differences.

Moreover, we further conducted a correlation analysis between insular neurobiomarkers and immunoendocrine markers to better elucidate the mechanism of dysregulated insular cortex activity and immunoendocrine status in the central–peripheral system interaction. The immune–endocrine axis is an important part of the pathogenesis of Graves’ disease and subsequent TED, with cellular and humoral immunity affecting the production status of thyroxine and other endocrine hormones [42]. Therefore, we detected major immunoendocrine markers in TED and performed a correlation analysis with the insular neurobiomarkers. Significant correlations were found between these two types of markers, further revealing the mechanism of central–peripheral system interaction in TED. In the inactive immunoendocrine status, the local activity of the insular cortex was correlated with the number of T and NK cells. These two types of cells were involved in the immunological pathogenesis of TED [43, 44], whose fluctuating level reflected abnormal immunoendocrine status in the peripheral system. In TED patients with active immunoendocrine status, the insula–cerebellum connectivity was positively correlated with the number of T cells and fT3 and fT4 levels. It is noteworthy that the negative correlation in Fig. 3B and C also indicated a positive association between the intensity of negative FC and fT3 and fT4 levels. This observation indicated that the severity of overactive thyroid was directly proportional to the degree of reduced connectivity between the insular cortex and the cerebellum. The correlation result between neuroimaging findings and clinical characteristics supported our major finding that dysregulated insular cortex activity was closely associated with the immunoendocrine status in TED. Since regulating immunoendocrine status poses significant challenges in managing autoimmune diseases like TED [8], the insular cortex might emerge as a target for innovative interventions in autoimmune diseases.

Although our preliminary study yielded valuable insights into the central–peripheral system interaction in TED, it had certain limitations. Firstly, the relatively limited sample size might have restricted the generalizability of our findings. Secondly, the levels of only the main neurobiomarkers and immunoendocrine markers were measured and analyzed, potentially omitting comprehensive assessments and understanding of the central–peripheral interface. Thirdly, we did not completely eliminate the vigilance effect caused by caffeine or other factors in the subjects by asking in advance. Finally, the cross-sectional nature of the study limited valuable insights into temporal dynamics, causal relationships, and mechanistic interpretations of dysregulated insular cortex activity and immunoendocrine status in the central–peripheral system interaction. More precise subtyping of TED based on immunoendocrine markers can be achieved and detailed longitudinal data can be obtained before and after immunosuppressive treatment for analysis in the future to further reveal the central–peripheral system interaction of TED.

### Supplementary Information

**Additional file 1.** **Material S1.** Participants. **Material S2.** Immunoendocrine status assessment. **Material S3.** Neuroimaging assessment. **Material S4.** Demographic, clinical characteristics, and immunoendocrine markers differences. **Material S5.** Discriminative features of ALFF. **Fig S1.** The altered amplitude of low-frequency fluctuations during the resting state in patients with thyroid eye disease with different immune statuses to controls. **Table S1.** Abnormal amplitude of low-frequency fluctuation in TED Patients. **Table S2.** Differences in functional connectivity among different immunoendocrine statuses of TED and healthy controls.

## Data Availability

The datasets utilized in the present study are not presently accessible to the public due to ongoing analysis. However, they can be obtained from the corresponding author upon making a reasonable request.

## Abbreviations

TED: Thyroid eye disease
AP: Active patients
IP: Inactive patients
HC: Healthy controls
ALFF: Amplitude of low-frequency fluctuation
FC: Functional connectivity
INS.R: The right insular cortex
rs-fMRI: Resting-state functional magnetic resonance imaging
EUGOGO: The European Group on Graves' Orbitopathy
CAS: Clinical activity score
CD: Cluster of differentiation
TSH: Thyroid-stimulating hormone
fT3: Free triiodothyronine
fT4: Free thyroxine
TRAb: TSH receptor antibody
NK cells: Natural killer cells
ANOVA: One-way analysis of variance
GRF: Gaussian random field
CER-6.L: Left cerebellum 6
CER-6.R: Right cerebellum 6
