# Supplementary material for: The insular cortex is not insular in thyroid eye disease: neuroimaging revelations of central–peripheral system interaction
Source: J Neuroinflammation. 2024 Feb 17;21:51. doi: 10.1186/s12974-024-03044-4 (PMC10874024; doi:10.1186/s12974-024-03044-4)
Supplement: Supplementary file 1 — Additional file 1. Material S1. Participants. Material S2. Immunoendocrine status assessment. Material S3. Neuroimaging assessment. Material S4. Demographic, clinical characteristics, and immunoendocrine markers differences. Material S5. Discriminative features of ALFF. Fig S1. The altered amplitude of low-frequency fluctuations during the resting state in patients with thyroid eye disease with different immune statuses to controls. Table S1. Abnormal amplitude of low-frequency fluctuation in TED Patients. Table S2. Differences in functional connectivity among different immunoendocrine statuses of TED and healthy controls. [file 12974_2024_3044_MOESM1_ESM.docx]

**Material S1.**

##### Participants

This prospective study has received approval from the Ethics Committee of Shanghai Ninth People’s Hospital, Shanghai Jiao Tong University School of Medicine (approval number: SH9H-2022-T229-2). Patients with TED and healthy controls (HC) were enrolled from December 2022 to March 2023. All subjects volunteered to participate in the study and gave their informed consent. A total of 116 TED patients, including 57 active patients (AP) and 59 inactive patients (IP) were recruited, along with 60 well-matched HC were included. TED was diagnosed according to the clinical guidelines proposed by the European Group On Graves' Orbitopathy (EUGOGO)(1). The participants of the three groups were matched in sex, age, and education level. Disease duration was determined from the onset of ocular manifestations. TED activity was assessed based on a combined consideration of the seven-point clinical activity score (CAS) and orbital MRI, according to the clinical guidelines (1,2). Exclusion criteria were: (1) signs and prior histories of various eye illnesses, e.g. amblyopia, cataracts, and glaucoma; (2) history of eye surgery; (3) history of psychiatric or neurologic illness, e.g., depression and bipolar disorder; (4) history of other endocrine diseases despite thyroid disorders; (5) anatomical abnormalities of the brain, e.g., tumors, trauma, and infection; (6) ineligibility for MRI scanning; (7) poor image quality that would impair the accuracy of fMRI analysis, e.g., motion artifacts, image distortion; (8) signs indicating DON (3).

**Material S2.**

##### Immunoendocrine status assessment

As aforementioned, TED patients were divided into two groups with different immune statuses (AP, IP) based on their disease activity according to a combined consideration of the seven-point clinical activity score (CAS) and orbital MRI. The AP group and IP group exhibit significant differences in immune status and display distinct immunomodulation patterns. The serum thyroid function tests, thyroid antibody tests, and lymphocyte subset analyses were performed by the clinical laboratory. The serum concentrations of thyroid-stimulating hormone (TSH), free triiodothyronine (fT3), and free thyroxine (fT4) were determined on a BECKMAN COULTER UniCel D×I800 immunoassay system. The TSH receptor antibody (TRAb) was measured by Cobas 6000 (Roche). The normal range of thyroid function: TSH: 0.56–5.91 uIU/ml; fT3: 3.1 - 6.8 pmol/L; fT4: 12.0 - 22.0 pmol/L; TRAb: 0.00–1.75 IU/L.

The lymphocyte subgroups were assayed by BD FACSCanto II flow cytometry system. Briefly, whole blood was used and staining for lymphocyte surface markers was performed after red cell lysis, according to a standard flow cytometric multicolor protocol and the manufacturer’s instructions. The CD3^+^ T cells, CD19^+^ B cells, and CD16^+^CD56^+^ NK cells were measured.

**Material S3.**

##### Neuroimaging assessment

For the neuroimaging acquisition, wakeful rs-fMRI was conducted on a 3T scan using a 64-channel phase array head coil (Magnetom Vida, Siemens, Erlangen, Germany). The entire brain was covered by high-resolution sagittal structural T1-weighted images and functional images. Head motion and scanning noise were reduced by using foam padding and earplugs. All subjects were required to close their eyes without dozing off when undergoing MRI scanning to reduce the interference of vigilance (4). The high-resolution sagittal structural T1-weighted images (3D-T1WI) for rs-fMRI, with the following parameters (TR = 2,400 ms, TE = 2.4 ms, thickness = 0.8 mm, gap = 0 mm, matrix = 320 × 320, FOV = 256 × 256 mm^2^, FA = 8°, number of slices = 208 and voxel size = 0.8 × 0.8 × 0.8 mm^3^) and functional images with the parameters (TR = 2000 ms, TE = 30 ms, thickness = 2 mm, matrix = 104 ×104, FOV = 208 × 208 mm^2^, FA = 90°, number of slices = 72, gap = 0 mm, and voxel size = 2 × 2 × 2 mm^3^) were obtained.

For neuroimaging data processing, DPABI version 7.0 (http://rfmri.org/DPABI) was used to preprocess all of the rs-fMRI data based on the MATLAB platform (www.mathworks.com/products/matlab)(5). For the purposes of magnetization balancing, the first 10 functional volumes were eliminated. Slice timing and realignment for head motion correction were performed. A linear registration algorithm was used to normalize the images to the Montreal Neurological Institute (MNI) EPI template (resampling voxel size = 3 mm × 3 mm × 3 mm), and a 6-mm full-width at half-maximum (FWHM) Gaussian kernel was used for smooth. Detrending was applied to remove linear trends. Finally, the nuisance covariates were eliminated using linear regression, including the average signals from the cerebrospinal fluid and white matter as well as the six head motion parameters. 3 participants were excluded from the final analysis because the maximal translational or rotational head movement exceeded 3.0 mm or 3.0^◦^. Moreover, there were no differences among the three groups (p = 0.1754) in framewise displacement (FD), and the mean FD of the AP, IP, and HC group is 0.17, 0.17, and 0.14, respectively (6). For the calculation of FC, head motion scrubbing regressors (FD threshold 0.2 for “bad” time) were also added in the linear regression to address the concern of motion (7–9).

The amplitude of low-frequency fluctuation (ALFF) and functional connectivity (FC) were computed across the whole brain. The time courses were converted to the frequency domain with a fast Fourier transform algorithm to enable each voxel to contain the amplitude of the signal across the whole spectrum. The averaged square root of the spectrum spanning the frequency range of 0.01-0.08 Hz was taken as the ALFF measurement. For standardized variability among the participants, the mean ALFF was obtained as the ALFF value divided by the global mean ALFF value.

After the ALFF analysis, the cluster of the insular cortex identified from the ALFF analysis was saved as seeds to further investigate the integration of the brain functional network via whole-brain FC analysis in a voxel-wise whole-brain manner. Fisher’s z transformation was then performed for normalization and statistical analysis facilitation.

**Material S4.**

##### Demographic, clinical characteristics, and immunoendocrine markers differences

Key demographic and clinical characteristics are presented in Supplementary Table 1. Three groups were matched in sex, age, and education level. The two patient groups showed no significant difference except for the CAS, which is a parameter reflecting their immunoendocrine statuses to some extent.

Immunoendocrine markers differences were shown in Table 2. There were significant differences in the levels of fT3, fT4, and TRAb between the two patient groups with varying immune statuses. However, there was no significant difference found in the counts of the cluster of differentiation. CD19^+^ B cells were higher in AP than IP, while TSH, CD3^+^ T cells and CD16^+^CD56^+^ NK cells were lower in AP, but no statistical difference was found. The levels of TRAb were significantly higher in AP compared to IP. However, the levels of fT3 and fT4 were significantly lower in AP.

**Material S5.**

##### Discriminative features of ALFF

The results of the one-way ANOVA analysis showed that the ALFF was significantly different in the right insular cortex (INS.R), the left inferior parietal lobule (IPL.L), and the right paracentral lobule (PCL.R). The ALFF in PCL.R was significantly higher in HC compared to TED. The ALFF in IPL.L was significantly higher in IP while the ALFF in INS.R was significantly higher in AP.

**References:**

1. Bartalena L, Kahaly GJ, Baldeschi L, Dayan CM, Eckstein A, Marcocci C, et al. The 2021 European Group on Graves’ orbitopathy (EUGOGO) clinical practice guidelines for the medical management of Graves’ orbitopathy. European Journal of Endocrinology. 2021 Oct 1;185(4):G43–67.

2. Oculoplastic and Orbital Disease Group of Chinese Ophthalmological Society of Chinese Medical Association, Thyroid Group of Chinese Society of Endocrinology of Chinese Medical Association. [Chinese guideline on the diagnosis and treatment of thyroid-associated ophthalmopathy (2022)]. Zhonghua Yan Ke Za Zhi. 2022 Sep 11;58(9):646–68.

3. Blandford AD, Zhang D, Chundury RV, Perry JD. Dysthyroid optic neuropathy: update on pathogenesis, diagnosis, and management. Expert Review of Ophthalmology. 2017 Mar 4;12(2):111–21.

4. Liu TT, Falahpour M. Vigilance Effects in Resting-State fMRI. Front Neurosci. 2020 Apr 23;14:321.

5. Yan CG, Wang XD, Zuo XN, Zang YF. DPABI: Data Processing & Analysis for (Resting-State) Brain Imaging. Neuroinform. 2016 Jul;14(3):339–51.

6. Jenkinson M, Bannister P, Brady M, Smith S. Improved Optimization for the Robust and Accurate Linear Registration and Motion Correction of Brain Images. NeuroImage. 2002 Oct;17(2):825–41.

7. Liu TT. Reprint of ‘Noise contributions to the fMRI signal: An Overview’. NeuroImage. 2017 Jul;154:4–14.

8. Yan CG, Cheung B, Kelly C, Colcombe S, Craddock RC, Di Martino A, et al. A comprehensive assessment of regional variation in the impact of head micromovements on functional connectomics. NeuroImage. 2013 Aug;76:183–201.

9. Satterthwaite TD, Elliott MA, Gerraty RT, Ruparel K, Loughead J, Calkins ME, et al. An improved framework for confound regression and filtering for control of motion artifact in the preprocessing of resting-state functional connectivity data. NeuroImage. 2013 Jan;64:240–56.

**
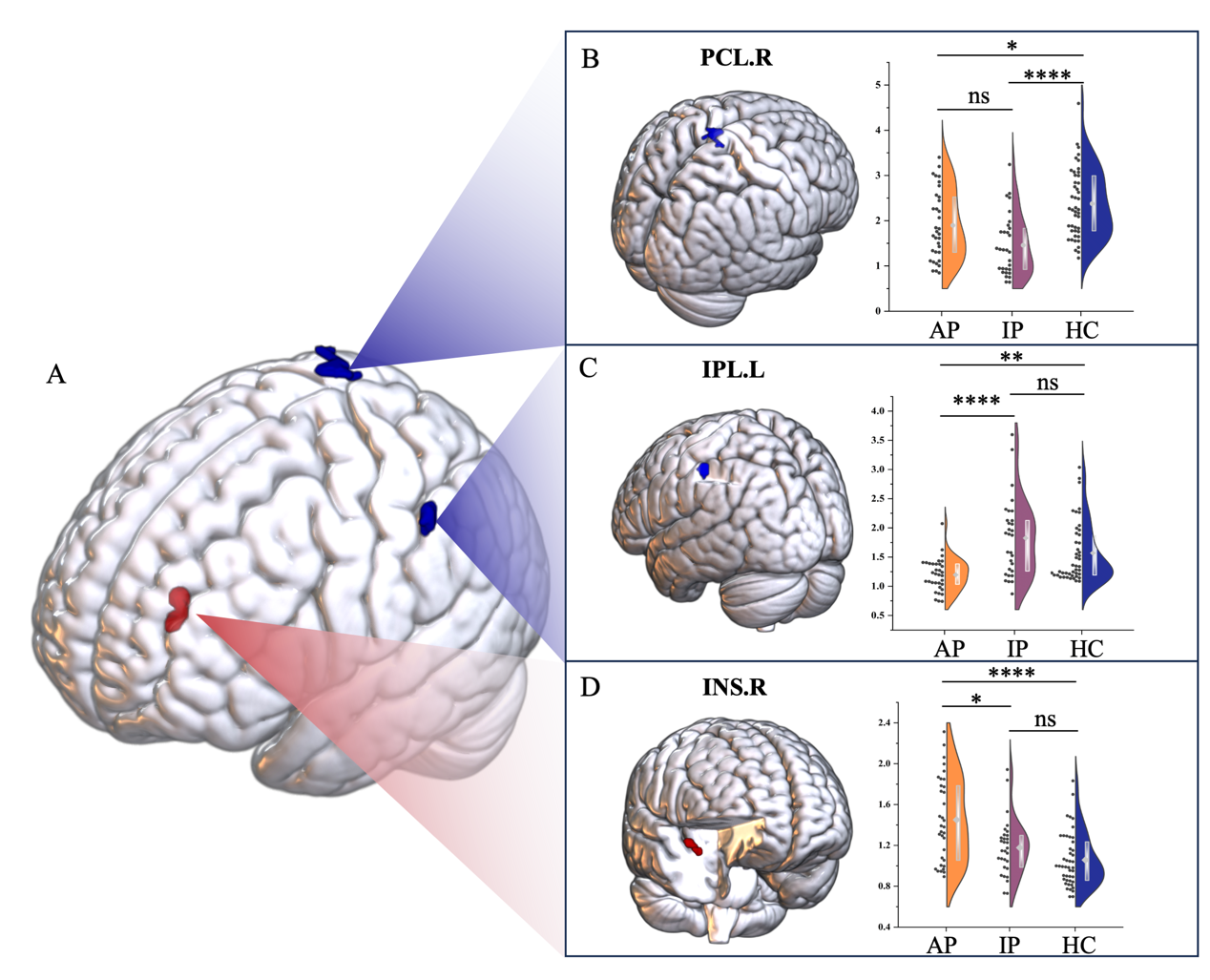
**

**Figure S1. The altered amplitude of low-frequency fluctuations during the resting state in patients with thyroid eye disease with different immune statuses to controls.** The differences primarily existed in the PCL.R, IPL.L, and INS.R (voxel *P* < 0.001, cluster *P* < 0.05, cluster-level GRF corrected). The red area represents a significantly activated brain region while the blue areas represent significantly inactivated brain regions. The violin and box plot demonstrates the ALFF differences of PCL.R, IPL.L, and INS.R in the AP, IP, and HC groups correspondingly.

ns *P* > 0.05; * *P* < 0.05; ** *P* < 0.01; **** *P* < 0.0001. AP, active patients; IP, inactive patients; HC, healthy controls; PCL.R, right paracentral lobule; IPL.L, left inferior parietal lobule; INS.R, right insular cortex.

| **Table S1.** Abnormal amplitude of low-frequency fluctuation in TED Patients | | | | | | | | |
| --- | --- | --- | --- | --- | --- | --- | --- | --- |
| Parameter | Conditions | Mask | Brain regions | F value | Cluster size | MNI coordinates of peak voxel | | |
|  |  |  |  |  |  | X | Y | Z |
| ALFF | TED < HC | Grey Matter | PCL.R | 14.1695 | 18 | 6 | -36 | 78 |
|  | AP < IP |  | IPL.L | 12.8055 | 11 | -51 | -33 | 48 |
|  | AP > IP |  | INS.R | 15.0464 | 14 | 42 | 0 | -9 |
| Abbreviations: TED: thyroid eye disease; AP: active patients; IP: inactive patients; HC: healthy controls; ALFF: amplitude of low-frequency fluctuation; PCL.R: right paracentral lobule; IPL.L: left Inferior parietal lobule; INS.R: right insular cortex; MNI: Montreal Neurological Institute. | | | | | | | | |

| **Table S2.** Differences in functional connectivity among different immunoendocrine statuses of TED and healthy controls | | | | | | | | | | | |
| --- | --- | --- | --- | --- | --- | --- | --- | --- | --- | --- | --- |
| Seed area | Seed coordinates | | | Mask | F value | Cluster size | Connected location | MNI coordinates of peak voxel | | |  |
| INS.R | 42 | 0 | -9 | Grey Matter | -4.906 | 73 | CER-6.R | 21 | -60 | -33 |  |
|  |  |  |  |  | -5.463 | 72 | CER-6.L | -15 | -66 | -24 |  |
| Abbreviations: TED: thyroid eye diseases; INS.R: right insular cortex; CER-6.R: Right cerebellum-6; CER-6.L: Left cerebellum-6; MNI: Montreal Neurological Institute. | | | | | | | | | | | |
